# AWGE-ESPCA: An edge sparse PCA model based on adaptive noise elimination regularization and weighted gene network for *Hermetia illucens* genomic data analysis

**DOI:** 10.1371/journal.pcbi.1012773

**Published:** 2025-02-13

**Authors:** Rui Miao, Hao-Yang Yu, Bing-Jie Zhong, Hong-Xia Sun, Qiang Xia

**Affiliations:** Basic Teaching Department, Zhuhai Campus of Zunyi Medical University, Zhu Hai, China; Max-Planck-Institute of Biochemistry: Max-Planck-Institut fur Biochemie, GERMANY

## Abstract

*Hermetia illucens* is an important insect resource. Studies have shown that exploring the effects of Cu^2+^-stressed on the growth and development of the *Hermetia illucens* genome holds significant scientific importance. There are three major challenges in the current studies of *Hermetia illucens* genomic data analysis: firstly, the lack of available genomic data which limits researchers in *Hermetia illucens* genomic data analysis. Secondly, to the best of our knowledge, there are no Artificial Intelligence (AI) feature selection models designed specifically for *Hermetia illucens* genome. Unlike human genomic data, noise in *Hermetia illucens* data is a more serious problem. Third, how to choose those genes located in the pathway enrichment region. Existing models assume that each gene probe has the same priori weight. However, researchers usually pay more attention to gene probes which are in the pathway enrichment region. Based on the above challenges, we initially construct experiments and establish a new Cu^2+^-stressed *Hermetia illucens* growth genome dataset. Subsequently, we propose AWGE-ESPCA: an edge Sparse PCA model based on adaptive noise elimination regularization and weighted gene network. The AWGE-ESPCA model innovatively proposes an adaptive noise elimination regularization method, effectively addressing the noise challenge in *Hermetia illucens* genomic data. We also integrate the known gene-pathway quantitative information into the Sparse PCA(SPCA) framework as a priori knowledge, which allows the model to filter out the gene probes in pathway-rich regions as much as possible. Ultimately, this study conducts five independent experiments and compared four latest Sparse PCA models as well as representative supervised and unsupervised baseline models to validate the model performance. The experimental results demonstrate the superior pathway and gene selection capabilities of the AWGE-ESPCA model. Ablation experiments validate the role of the adaptive regularizer and network weighting module. To summarize, this paper presents an innovative unsupervised model for *Hermetia illucens* genome analysis, which can effectively help researchers identify potential biomarkers. In addition, we also provide a working AWGE - ESPCA model code in the address: https://github.com/yhyresearcher/AWGE_ESPCA.

## Author summary

*Hermetia illucens* is an insect of high economic value, which is widely used in the field of feed. Existing research suggests that Cu^2+^-stressed can significantly affects the growth of *Hermetia illucens*. Therefore, the identification of genetic target information affecting the growth and development of *Hermetia illucens* is crucial for food safety. However, due to the lack of high-quality data sets, high data noisy and low sample number. None of the existing genomic analysis models can handle the *Hermetia illucens* data well. Based on the above problems, a novel unsupervised *Hermetia illucens* genomic analysis model (AWGE-ESPCA) is proposed in this paper. The AWGE-ESPCA model proposes a daptive noise elimination regularization to solve noise challenges in data and uses weighted gene network to enhance the biological interpretability capability of the model. The experimental results show that the AWGE-ESPCA model can well select potential target genes and key pathways. In addition, we demonstrate that the AWGE-ESPCA model can be extended to other insect genome analysis tasks.

## 1. Introduction

*Hermetia illucens* is a globally important resource insect that plays a great role in many fields [1–3]. For example, *Hermetia illucens* larvae feed on decaying organic matter and animal feces, and are widely used in various countries in the field of environmentally sound treatment. In addition, its by-products are rich in nutrients and antimicrobial peptides, which have a wide range of applications in the fields of feed and medicine [4,5]. Studies have shown that high concentration of Cu^2+^-stressed not only seriously affects the growth and development of *Hermetia illucens* [6,7], but also reduces the content of nutrients such as total sugars and proteins in the hemolymph of the larvae [6,7], thus decreasing its value in feed and medicine. More importantly, if the concentration of Cu^2+^-stressed in the feed of *Hermetia illucens* is too high, it may also poses a risk to human beings through bioconcentration [8,9]. Therefore, studying the effects of Cu^2+^-stressed in the growth and development of *Hermetia illucens* can improve its application value in environmental harmless treatment, animal feed safety and medicine [10–12].

Currently, there are three major challenges for the Cu^2+^-stressed *Hermetia illucens* genome analysis [13]: Firstly, there are few available data on Cu^2+^-stressed *Hermetia illucens* [14]. Although some genomic datasets of Cu^2+^-stressed *Hermetia illucens* growth have been constructed by researchers [14,15]. However, the sample sizes of the existing datasets are very small, and no researchers have constructed genomic datasets of *Hermetia illucens* under different Cu^2+^-stressed environments. It prevents researchers from building high-performance models for *Hermetia illucens* genomics analysis. Secondly, to the best of our knowledge, there are still no AI feature selection models specifically for insect genomes. The noise problem of insect genomic data is even more serious compared to human genomic data [13,16]. In the case of *Hermetia illucens* data, the dataset contains many gene probes with excessive expression values. These gene probes are not relevant to sample classification but can significantly image the feature selection results of the AI model. Therefore, it is essential to construct an AI feature selection model specifically designed to analyze insect genomic data and address the noise challenge of the data. Thirdly, choose those genes located in the pathway enrichment region. The existing models assume that each gene probe has the same priori weight. Based on biological common sense, it is obvious that researchers will be more concerned with regions that are more enriched in biological pathways [17,18]. In other words, gene probes that are associated with a larger number of gene pathways should obviously receive more attention. This is because these gene probes may play more important roles in the upstream and downstream processes of biological actions [19]. Therefore, how to rationally weight the gene probes in the model based on the known information about the number of pathways, so as to filter out the pathway-enriched regions is the third major challenge currently faced.

**Fig 1:**
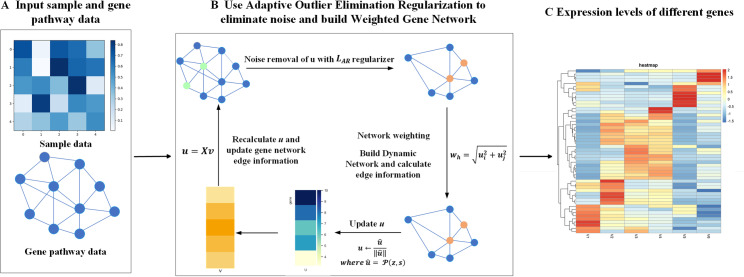
The algorithm of the AWGE-ESPCA model. The steps are as follows: (A) Input Data: It includes sample data and gene pathway data. (B) Data Processing: It involves two core modules. First, Adaptive Noise Elimination Regularization is used to eliminate noise; then, Weighted Gene Network is constructed. (C) Result Output: It obtains gene expression values with different weights.

Based on the above challenges, we design experiments and build a Cu^2+^-stressed *Hermetia illucens* genomics dataset. Then, we propose AWGE-ESPCA: An Edge Sparse PCA Model Based on Adaptive Noise Elimination Regularization and Weighted Gene Network (Fig 1). AWGE-ESPCA proposes an Adaptive Noise Elimination Regularizer for solving the noise problem of *Hermetia illucens* genome data. The AWGE-ESPCA model also uses the known gene-pathway quantity information as a priori knowledge and integrates it into the gene network of the Sparse PCA model. This weighted gene network allows the model to pay more attention to the pathway-rich regions in the gene network, and these gene probes are often the key sites in the pathway.

In this paper, we conduct five independent experiments. Firstly, we set up a noisy dataset for simulation experiments to verify the noise resistance of AWGE-ESPCA. The results show that AWGE-ESPCA can still accurately identify target genes even in the presence of noise. Secondly, we conduct experiments in the *Hermetia illucens* dataset. The experimental results show that the AWGE-ESPCA model is superior to existing models. Pathway enrichment analysis shows that AWGE-ESPCA model can effectively select the pathway information related to growth and development. Thirdly, we select a public insect dataset for our experiments, which shows that the AWGE-ESPCA model can be generalized to analyze data from other insects as well. Fourthly, we perform ablation experiments to validate the role of each component for AWGE-ESPCA. Finally, we perform bioenrichment analyses to identify biomarkers that might influence *Hermetia illucens* growth.

## 2. Related work

In terms of dataset construction, researchers have created a few datasets on the connection between Cu^2+-^stressed and the growth and development of *Hermetia illucens*. For instance, Wang et al. researches the concentration of metal ions in the feces of larvae of *Hermetia illucens* grown under different concentrations of Cu^2+^-stressed [6]. This experiment provides compelling evidence that *Hermetia illucens* will be enriched in excess Cu^2+^-stressed, which will impact the larvae’s growth and development. Deng et al. [20] constructs experiments to verify that Cu^2+^-stressed and Cu^2+^-stressed directly affect the body weight and pupation rate of *Hermetia illucens* larvae. However, no researchers have established genomic datasets of *Hermetia illucens* growth under different concentrations of Cu^2+^-stressed. This prevents the researchers from studying the important genes and pathways affected by Cu^2+^-stressed on the growth of *Hermetia illucens*, and how these genes might affect poultry and humans as they become enriched.

In genome analysis models, the common machine learning models to study the genome analysis of insects can be divided into two categories currently [21,22]. One category is supervised learning models [21]. Theoretically, supervised learning models have high prediction accuracy and good performance, but the model needs a lot of samples for training [22,23]. For insect data, the sample size is relatively limited [24]. Therefore, although supervised learning models perform well in many specific genomic data analysis tasks [25], it is difficult to effectively analyze insect genomic data. Another category is unsupervised learning models [26]. The advantages of unsupervised learning models include low sample size requirements, the ability to learn from unlabeled data and discover underlying patterns and structures [27,28]. They are excellent in processing high-dimensional data, dimensionality reduction, clustering and outlier detection [29,30]. For insect data with a relatively limited sample size, unsupervised learning models show greater potential and advantages in insect genome analysis [31]. Among the unsupervised models, the PCA model is one of the most commonly used models for dimensionality reduction and feature extraction [32,33]. However, the traditional PCA model arithmetic process is not able to remove the noise contained in the high-dimensional gene data [34], which reduces the credibility of the PCA model results. Therefore, some researchers proposes the Sparse PCA model (SPCA) [35], which introduces the $L_{0}$ regularizer to remove the noise in the data, characterized by strong biological interpretability and fast operation speed. The SPCA model achieves good performance in many biological application scenarios [36,37]. Although the SPCA model has a great improvement in the ability of noise removal and dimensionality reduction analysis compared with the PCA model, the SPCA model is not able to utilize the known biological network structure information [36]. In 2018, Min et al. [38] proposed the Sparse Edge Group PCA model (ESPCA) for feature extraction of gene data. In 2022, Miao et al. [39] proposed a Sparse PCA model based on meta-learning and dynamic networks (DM-ESPCA) to further improve the feature selection ability of the Sparse PCA model. However, the existing Sparse PCA models are still not a good solution to the noise problem in insect datasets. Since the existing Sparse PCA models are based on the $L_{0}$ regularizer for solving [40], the models will almost certainly select gene probes with too large expression values even if these gene probe are not related to the target of the experiment. Finally, all current Sparse PCA models assume that all gene probes have the same priori information in the pathway, which is also clearly not in line with biological common sense. Researchers usually prioritize those gene probes that are located in pathway-enriched regions [17,18]. This is because these gene probes may be the key probes that influence the epigenetic characteristics of organisms.

## 3. Materials and models

### 3.1. Ethics statement

The experimental populations were maintained under standard laboratory conditions following established protocols for insect welfare. All Cu^2+^-stressed treatments were performed within approved safety guidelines for heavy metal exposure studies.

The RNA-seq experiments were performed by BGI Shenzhen Co., Ltd. in compliance with standard biosafety protocols and quality control procedures. The Drosophila melanogaster dataset (GSE243439) was obtained from the Gene Expression Omnibus (GEO) database and used in accordance with their data usage policies. The pathway information for Hermetia illucens was provided by Shenzhen UW Genome Science and Technology Service Co. under appropriate data sharing agreements.

### 3.2. Dataset

#### 3.2.1. Hermetia illucens datasets.

***3.2.1.1. Experimental design and population establishment*:** First, we establish an experimental population of *Hermetia illucens* under copper ion (Cu^2+^) stressed. *Hermetia illucens* eggs are placed in a plastic rearing box in an artificial climate chamber for incubation. Following hatching, we begin raising the larvae by providing artificial diet initially, but without the addition of Cu^2+^-stressed. Upon reaching 4 days of age, they are divided into groups for Cu^2+^-stressed experiments. To create experimental populations under Cu^2+^-stressed, artificial feeds with varying Cu^2+^-stressed concentrations (0 mg/kg, 75 mg/kg, 150 mg/kg, 300 mg/kg, 600 mg/kg, and 1,200 mg/kg) are fed to the groups for five generations. Three replicates are set up for each treatment group.

***3.2.1.2. High-throughput sequencing*:** Total RNA is isolated from the 5th instar larvae of *Hermetia illucens* in each treatment group using Trizol reagent (Invitrogen, Carlsbad, CA, USA). The purity and concentration of the RNA samples are determined using a Nanodrop spectrophotometer and a Qubit® 2.0, and the integrity of the RNA samples is determined using an Agilent 2100. After the total RNA samples pass the inspection, the RNA of *Hermetia illucens* larvae from each treatment group is sent to BGI Shenzhen Co., Ltd. for transcriptome sequencing using the Illumina Hiseq4000 sequencing platform. The sequencing results are RNA-seq expression values, with each sample analyzed for expression levels across 19,512 gene probes.

#### 3.2.2. Drosophila melanogaster dataset.

A dataset from the Gene Expression Omnibus (GEO) database is used this time: GSE243439 [https://www.ncbi.nlm.nih.gov/geo/query/acc.cgi?acc=GSE243439].

The core goal of this dataset is to find genes associated with the development of Chronic Myeloid Leukaemia (CML) in Drosophila melanogaster, with nine samples.

These nine samples are divided into three groups: the first is a control (W1118), the second is BCR-ABLp210, and the third is BCR-ABLT315I. The control (W1118) is a normal Drosophila, whereas BCR-ABLp210 and BCR-ABLT315I represent two different forms of BCR-ABL1 fusion proteins respectively.

The BCR-ABL1 fusion protein is produced by the fusion of the BCR and ABL1 genes and has aberrant tyrosine kinase activity, leading to CML. BCR-ABLp210 is the common form of the fusion protein, whereas BCR-ABLT315I is a drug-resistant mutant form.

Among them, we choose the control group and the BCR-ABLp210 group for our study because BCR-ABLp210 is the main causative gene of CML, and the study of its molecular characterisation can provide basic data that are more widely applicable to most CML patients.

#### 3.2.3. Gene pathway data sets.

Pathway information for *Hermetia illucens* gene is provided by Shenzhen UW Genome Science and Technology Service Co. Total 65829 pathway information. The Pathway of Drosophila melanogaster dataset comes from Pathway Commons dataset at http://www.pathwaycommons.org/ and https://www.genome.jp/kegg/.

### 3.3. Models

#### 3.3.1. SPCA.

Suppose there is an existing gene matrix $X\in R^{m,n}$, where *m* denotes the number of gene probes and *n* denotes the number of samples. The researcher usually uses Singular Value Decomposition (SVD) framework to solve genome sparsification problems based on $L_{0}$ regularizer. The optimization problem is formulated as equation (1):


$$\underset{∥\text{u}∥{\;}_{\text{2}}\;\leq \text{1,}∥\text{v}{∥}_{\text{2}}\leq \text{1}}{\text{maximize}}\;{\text{u}}^{\text{T}}\text{Xv, s}\text{.t}\text{.}∥\text{u}{∥}_{\text{0}}\leq \text{s}$$ (1)

where *u* is an m×1 vector represents the first principal component (PC) loadings, while *v* represents the corresponding n×1 PC. The value of *s* represents the count of genes that the model retains. The $∥u∥{\;}_{0}\;$ and $∥u∥{\;}_{2}\;$represent the $L_{0}$ and $L_{2}$ norms. Since the $L_{0}$ norm is not directly minimizable due to its non-convex nature, researchers generally adopt a greedy principle for the solution, i.e. $∥u∥{\;}_{0}=P\left(z,\;s\right)$,where $P\left(z,s\right)$ achieves sparse projection. For vector *u*, its *k* -th entry is defined by equation (2):


$${\left[\text{P}\left(\text{z},\text{s}\right)\right]}_{\text{k}}= \{\begin{array}{c} {\text{z}}_{\text{k}},\text{ if}\;k\in supp\;\left(z,s\right)\\ 0,\text{ otherwise } \end{array}$$ (2)

where $supp\left(z,s\right)$ denotes the set of indexes of the largest *s* absolute element of z.

#### 3.3.2. ESPCA.

In a 2018 study, Min et al. introduced the edge group Sparse PCA (ESPCA) [18]. The ESPCA framework evolves from the conventional probe sparse to a group sparse configuration, significantly enhancing the capability of feature discrimination in Sparse PCA. Let’s define *G* as a group structure within the gene interaction network, where two connected genes form a group. Obviously, such edge-groups are overlapping. We denote $G=\left\{e_{1},\ldots ,e_{m}\right\}$as an edge set with all edges from a given gene interaction network. Here, The ESPCA formulation is presented as equation (3):


$$∥u∥{\;}_{ES}\;=\underset{\forall G^{\prime }\in G,\;support\;\left(u\right)\;\subseteq \;V\left(G^{\prime }\right)}{minimize}\;\left|G^{\prime }\right|$$ (3)

where *G*^′^ is a subset of *G*, $V\left(G^{\prime }\right)$ is a vertex (gene) set induced from the edge set *G*^′^, $\left|G^{\prime }\right|$ denotes the number of elements of *G*^′^, and $support\left(u\right)$ denotes the set of indexes of nonzero elements of *u* [41]. Drawing from equation (4), this sparse model can be delineated as equation (4):


$$\underset{∥u∥{\;}_{2\;}\leq \;1,\;∥v∥{\;}_{2}\;\leq \;1}{maximize}\;u^{T}Xv,\;s.\;t.∥u{∥}_{\;ES}\leq s$$ (4)

where *s* represents the count of edges. The model also uses the greedy principle for sparse solving.

#### 3.3.3. AWGE-ESPCA.

**Fig 2:**
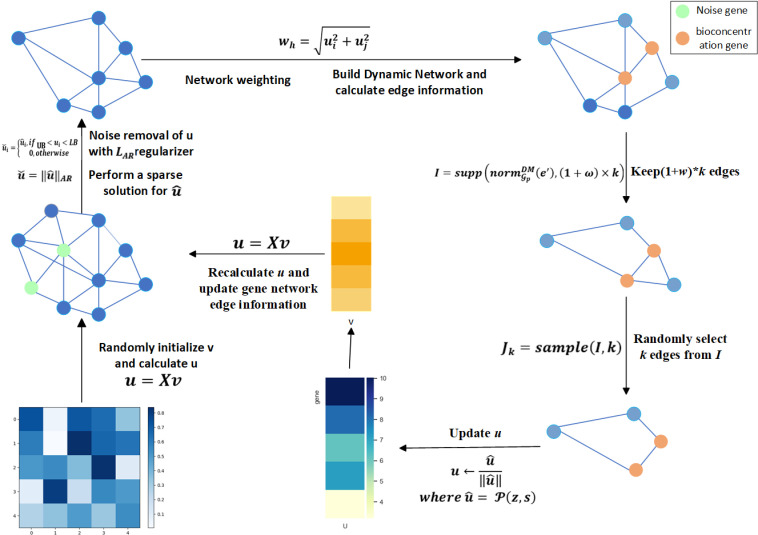
Flow chart of the AWGE-ESPCA model. Fig 2 shows the specific flow of the AWGE-ESPCA algorithm. First, randomly initialize v  and calculate *u*; then, identify $L_{A\text{R}}$ based on the modified regularizer and remove the data noise to get the new $\overset{˘}{u}$; then, calculate the edge information based on the gene network and the weighted information; finally, retain the important genes and edge information based on the $\overset{˘}{u}$ and edge information and continue to loop through the process based on the current result.

Here, we propose the AWGE-ESPCA model (Fig 2). Compared with the existing models, the AWGE-ESPCA model has two main improvements. First, the AWGE-ESPCA model proposes a new adaptive Noise Elimination regularizer. The noise problem in insect datasets is manifested as extremely uneven data distribution, with some data probes having extremely large values, while others have extremely small values. This data noise greatly reduces the credibility and biological interpretability of the algorithm and increases the complexity of data analysis. The adaptive Noise Elimination regularizer proposed in this paper can adaptively remove outliers from the data. It is more efficient and accurate than the previous manual selection of data. Second, the AWGE-ESPCA model proposes a new Weighted Gene Interaction Network (WGIN) module, which allows the model to focus on selecting key genes related to the pathway enrichment and target experiment function region. For example, the main goal of our *Hermetia illucens* gene data analyses is to find the pathway region related to growth and development, so WGIN’s weighting module will make the model pay more attention to the corresponding pathway region.

***3.3.3.1. Adaptive regularization for noise probes elimination*:** Firstly, the model will randomly initialize the sample weight feature vector *v*. Next, the gene probe weight feature vector u is calculated based on equation (5):


u=Xv  (5)

here, the weight $u_{i}$ of gene probes with excessive gene expression values will be very large, and we believe that they should be considered as potential noise in the data. Therefore, we propose the $L_{AR}$ regularizer to perform noise removal for *u*. The specific steps are shown in equations (6)–(12).

To begin, we sort *u* from smallest to largest on equation (6):


$$\hat{u}=\text{sort }\left(u\right)$$ (6)

Next, we compute the parameters $Q1_{\text{sorted}}$, $Q3_{\text{sorted}}$ according to equations (7) and (8):


$$\text{Q}1_{\text{sorted}}={\text{ u}}_{\text{k}},\text{ k}=\;∥\text{m}\text{*}\text{lq}∥{\;}_{\text{in}}$$ (7)

$$Q3_{\text{sorted}}=\;\;u_{k},\;k=\;∥m\;*\;\left(1-lq\right)\;∥{\;}_{in}$$ (8)

where lq is the regularization parameter of the model input, within the range (0, 0.5), and $∥*{∥}_{in}$_ in denotes the integerized regularizer, which is obtained by rounding up to the next integer if the decimal portion of $m*\left(1-lq\right)$ is greater than or equal to 0.5, otherwise it is rounded down to the current integer. Next, we calculate the upper edge weight parameter LB and the lower edge weight UB on equations (9) and (10):


$$\text{LB}=Q1_{\text{sorted }}-\left(1.5\;\times \text{IQR}\right)$$ (9)

$$\text{UB}=Q3_{\text{sorted }}+\left(1.5\;\times \;IQR\right)$$ (10)

where, $IQR=Q3_{\text{sorted }}-Q1_{\text{sorted }}$*.*

Finally, we sparsely solve for $\hat{u}\;$ using the sparse projection of equation (11), with:


$${\overset{ˇ}{u}}_{i}= \{\begin{array}{c} {\hat{u}}_{i},\;\;if\;UB<u_{i}<LB\\ 0,\;otherwise \end{array}$$ (11)

That is, the $L_{AR}$regularizer can be finally expressed as equation (12):


$$\overset{ˇ}{u}=\;∥\hat{u}∥{\;}_{AR}$$ (12)

***3.3.3.2. Weighted gene interaction network*:** After computing $\overset{ˇ}{u}$, we restore the order of the gene probes in $\overset{ˇ}{u}$ to the initial state based on their original positions in the vector *u*, $\overset{ˇ}{u}\;=\;returne\;\left(\overset{ˇ}{u}\right)$Next, we introduce the weighted gene network sparsification module as shown in equations (13)–(22):

We assume that $e_{h}=\left(u_{i},\;u_{j}\right)\in \;G,\;u_{i},\;u_{j}\in R^{m}$, and the weight $w_{h}\;$ of $e_{h}$ is define as formula (13):


$$w_{h}=\sqrt{u_{i}^{2}+u_{j}^{2}}$$ (13)

where $u_{i}$ and $u_{j}$ are the left and right gene probes of $e_{h}$.

First, we calculate a weight vector $Wq=\left\{wq_{1},\ldots ,wq_{m}\right\}$ for each gene probe based on the known gene-pathway relationship information, where $wq_{i}\;$ represents the number of pathways associated with the *i* -th gene probe that are relevant to the experiment target. In the absence of an explicit experimental goal, $wq_{i}$ represents the number of pathways corresponding to the *i* -th gene probe. For example, in the *Hermetia illucens* dataset, Wq  represents the number of gene pathways related to growth and development. Since the number of pathways for each probe in Wq  varies greatly, we normalize *W* to $\left[\text{a}-\text{b}\right]$ using equations (14) and (15):


$$k=\frac{b-a}{\text{Max}-\text{Min}}$$ (14)

$$wq_{i}=a+k\times \left(wq_{i}-\text{Min}\right)$$ (15)

where a,  b are the input parameters, Max = Max{W}, Min = Min{W}The edge weights of the *h*−th gene network can be represented as equation (16):


$$w_{h}=\sqrt{wq_{i}u_{i}^{2}+wq_{j}u_{j}^{2}}$$ (16)

Finally, we can get the edges set of the gene network $G\left(i\right)$. We use the Greedy-based Random Sampling Algorithm as proposed by DM-ESPCA for sparse solving of $G\left(i\right)$. That is, Algorithm 1. $P\left(z,k\right)$ is sparse projection, $\;{\left[P_{G}\left(z,k\right)\right]}_{i}\left(i=1,\ldots ,m\right)\;$ meets the condition in equation (17):


$${\left[P_{G}\left(z,k\right)\right]}_{\;i}= \{\begin{array}{c} z_{i},\;\;if\;G\left(i\right)\cap sample\left(I,k\right)\neq \emptyset \\ 0,\;otherwise \end{array}$$ (17)

where $I=supp\left(norm_{G}^{AW}\left(e^{\prime }\right),\;\left(1\;+\;\omega \right)\;\times \;k\right)$. If gene *i* is selected, ${\left[P_{G}\left(z,k\right)\right]}_{i}=z_{i}$, otherwise ${\left[P_{G}\left(z,k\right)\right]}_{i}=0$. *k* represents the number of edges expected to be retained. *ω* is a parameter that controls the random ratio.

Finally, we use formula (18)–(20) to update vectors *u* and *v* until the algorithm convergence:


$$u=Xv,\;where\;\hat{v}=X^{T}u\;$$ (18)

$$u\leftarrow \frac{\hat{u}}{∥\hat{u}∥},\text{ where }\hat{u}=P_{G}\left(z,k\right)\text{ and}\;z=Xv$$ (19)

$$v\leftarrow \frac{\hat{v}}{∥v∥},\text{ where }\hat{v}=X^{T}u$$ (20)

Ultimately, the AWGE-ESPCA model can be expressed as equation (21):


$$\underset{\;∥u∥{\;}_{2\;}\leq 1,\;∥v∥{\;}_{2}\leq 1}{maximize}u^{T}Xv,\;s.\;t.∥u∥{\;}_{AW}\leq s$$ (21)

**Algorithm 1: The algorithm of AWGE-ESPCA**


$$Require:\;X\in {\mathbb{R}}^{m\times n},\nu \in {\mathbb{R}}^{n\times 1},\;parameter\;k,\;\omega ,\;\rho ,$$

$$edge\;\;set\;\;G_{P}=\left\{e_{1},e_{2},\cdots e_{n}\right\}$$

1:Z=Xv #Randomize the initial *v* and compute *Z*
$2:Z=\;∥Z{∥}_{AW}$ #Adaptive Regularization for Noise Elimination

$3:Let\;norm_{G_{P}^{'}}^{AW}\left(e^{\prime }\right)={\left(∥e_{1}^{'}∥,\;\cdots ,∥e_{n}^{'}∥\right)}^{T}$

$4:for\;any\;weight\;of\;edge\;e\;\;\;in\;G_{P}\;doqq$

$5:w_{n}^{'}=\sqrt{wq_{i}z_{i}^{2}+wq_{j}z_{j}^{2}}\;\#Generate\;\text{WGIN}\;network.$

$6:\;update\;\;G_{Pn}^{'}=\;w_{n}^{'}$

7:end for

$8:I=supp\;\left(norm_{G_{p}}^{DM}\left(e^{\prime }\right),\;\left(1+\omega \right)\;\times k\right)\#Extract\;\;\left(1+\omega \right)\;\times \;k\;edges.$

$9:J_{k}=sample\;\left(I,k\right)\;\#Randomly\;\;select\;\;k\;\;edges\;\;from\;I.$

10:if  ω>0  then  ω= ω− ρ #Reduce  random  rate

$11:V_{\;G_{P}^{'}}=V\left(G_{P}^{'}\right)$

$12:for\;\;any\;gene\;\;i\;\;in\;\;V_{\;G_{P}^{'}}\;do$

$13:{\hat{u}}_{i}=z_{i}\#Determine\;\;the\;\;sparse\;\;u$

$14:end\;\;for\;14:u=\frac{\hat{u}}{∥\hat{u}∥}$

$15:return\;\;u\;\;and\;\;P_{G_{P}}\left(z,k\right)=\;\hat{u}$

## 4. Results

In total, we conduct comprehensive comparisons between AWGE-ESPCA and multiple methods, including traditional Sparse PCA variants (PCA, SPCA, ESPCA, DM-ESPCA), advanced dimensionality reduction methods (t-SNE, UMAP, AEs, VAEs), and supervised methods (Lasso, Elastic Net). For supervised methods, given the small sample size (6 samples), we utilize all samples as training data with Lasso (α = 0.01) and Elastic Net (*α* = 0.01, $l_{1\;}\_ratio$ = 0.5). All Sparse PCA methods calculate the first two PCs.

The first experiment is a simulation experiment. We generate a batch of carefully designed simulation data. The final results are to prove that the existing methods can not perform feature selection well in the face of strong noise. The second experiment is conducted based on the *Hermetia illucens* dataset. The experiments are divided into two steps. First, the *Hermetia illucens* dataset is distinctly divided into two groups based on the FPKM((Fragments Per Kilobase of transcript per Million mapped reads, a normalized measurement of gene expression level) values of copper ions, one group is low concentrations, including Cu_0_FPKM, Cu_75_FPKM, Cu_150_FPKM and Cu_300_FPKM, the other is high concentrations, including Cu_600_FPKM and Cu_1200_FPKM. The second step is to construct experiments based on different models. The goal is to screen the gene probes and pathway information that may affect the growth and development of *Hermetia illucens*. The third experiment is built on the Drosophila dataset with the aim of further verifying whether AWGE-ESPCA can be extended to the analysis of other insect genomic data. The fourth experiment is an ablation experiment. On the basis of *Hermetia illucens* dataset, we conduct two independent ablation experiments, which independently remove the $L_{AR}$ regularizer and Weighted Gene Interactive Network to validate the effect of the model. The fifth experiment is enrichment analysis, where we perform bio-enrichment analysis on the *Hermetia illucens* dataset to identify potential key pathway information.

To validate the performance of the model, we calculate five independent metrics, including: Heatmap (Top-50, Sparse PCA model using PC1), Sample Distribution Plot (based on PC1 and PC2), Number of Pathways (Top-500, Sparse PCA model using PC1), Target Gene Percentage (genes/total number of genes with experimental target function) and Box Plots of Gene Probe Expression (Top-100, Sparse PCA model using PC1). Among them, the heatmap and sample distribution map allow us to intuitively see whether the gene probes selected by each method can clearly distinguish the samples. The number of pathways and the distribution of target genes can be used to compare the biological feature selection capabilities of the models. The box plots of gene probe expression allow us to intuitively see the number of outliers and the range of average expression values contained in the gene probes selected by the model. This will validate the conjecture of this paper about data noise, that is the existing methods may select more gene probes containing larger expression values. In addition, since the T-SNE and UMAP models do not have feature selection capabilities, only a sample distribution plot comparison is performed for the T-SNE and UMAP models. The Lasso and Elastic Net models do not have dimensionality reduction capabilities, so no Sample Distribution Plot is drawn.

### 4.1. Simulation study

In this section, we construct an example to illustrate how it addresses the limitations inherent in the existing Sparse PCA model. We generate a simulated gene expression matrix *X* and an interaction network defined by an edge set *G*.

#### 4.1.1. Principal component (PC) loadings generation.

Two principal component loadings are defined as equations (22) and (23):


$$u_{1}={\left[1,\;\;0.86,\;\;0.66,\;\;0.9,\;,\;{\left[\text{rep}\left(0,8\right)\right]}^{T}\right]}^{T},$$ (22)

$$u_{2}={\left[{\left[\text{rep}\left(0,4\right)\right]}^{T},\;0.2,\;\;-0.55,\;\;-0.35,\;\;0.17,\;{\left[\text{rep}\left(0,4\right)\right]}^{T}\right]}^{T}$$ (23)

where $rep\;\left(0,\;a\right)$ denotes a column-vector of size *a*, with each element being zero.

#### 4.1.2. Principal components generation.

Two principal components are generated using the formulas in equation (24):


$$v_{1}=\text{rnorm}\left(100\right),v_{2}=\text{rnorm}\left(100\right)$$ (24)

here, $rnorm\left(b\right)$ represents a column-vector of size *b*, with elements randomly sampled from a standard normal distribution.

#### 4.1.3. Expression matrix construction.

The expression matrix $X\in R^{100\times 12}$ is constructed for 100 genes across 12 samples. Among them, the first 8 gene probes are the gene probes contained in PC1 and PC2. The last four gene probes are noisy gene probes, which are not associated with any PC but have large expression values (200–300), denoted as $\gamma 0=x^{100\times 4}$We generate these four gene probes to validate whether the model can remove the noise information from the data better. Finally, the simulated data expression matrix is represented as equation (25):


$$X=d_{1}u_{1}v_{1}^{T}+d_{2}u_{2}v_{2}^{T}+\gamma 0+\gamma \epsilon$$ (25)

where $d_{1}u_{1}v_{1}^{T}$ represents contributions related to PC1, and $d_{2}u_{2}v_{2}^{T}$ corresponds to those from PC2. γϵ stands for random perturbation.

#### 4.1.4. Expression matrix construction.

We have constructed a genetic network $\;y\;=\;y_{1}+\;y_{2}$, where $y_{1}$ and $y_{2}\;$ are as equations (26) and (27):


$$y_{1}=\left\{\left(1,\;\;2\right),\;\;\left(1,\;\;3\right),\;\left(1,\;4\right),\;\;\left(2,\;\;3\right),\;\;\left(2,\;\;4\right),\;\;\left(3,\;\;4\right),\;\;\left(5,\;\;9\right),\;\;\left(6,\;\;10\right)\right\}\;\;$$ (26)

$$y_{2}=\left\{\left(5,\;\;6\right),\;\left(5,\;\;7\right),\;\;\left(5,\;\;8\right),\;\;\left(6,\;\;7\right),\;\;\left(6,\;\;8\right),\;\;\left(7,\;\;8\right),\;\;\left(1,\;\;11\right),\left(3,\;12\right)\right\}$$ (27)

The experimental results strongly demonstrate the performance of AWGE-ESPCA model. Among the five methods, only the AWGE-ESPCA model successfully selects all the correct gene probes, while the remaining methods all select a large number of noisy gene probes. Especially the SPCA model, which selects all four noisy gene probes as PC1 and PC2 (Table 1 and Table A in S1 Text). The results of the simulation experiments confirm our conjecture that gene probes with excessive expression values significantly affect the results of the model in a high-noise environment.

**Table 1:** The top two identified PC1 and PC2 loadings by ESPCA, DM-ESPCA and AWGE-ESPCA.

Method	ESPCA		DM-ESPCA		AWGE-ESPCA	
**PC**	PC1	PC2	PC1	PC2	PC1	PC2
**Var1**	−0.001	−0.006	0	0.002	0.596	0
**Var2**	0	0	0	0	0.508	0
**Var3**	0	0.028	−0.001	0.028	0.422	0
**Var4**	0	0	0	0	0.458	0
**Var5**	0	0	0	0	0	0.294
**Var6**	−0.002	−0.052	−0.002	−0.046	0	−0.933
**Var7**	0	0	0	0	0	−0.071
**Var8**	0	0	0	0	0	0.195
**Var9**	−0.568	0	−0.571	0	0	0
**Var10**	−0.587	−0.525	−0.591	−0.333	0	0
**Var11**	−0.577	0.531	0	0.885	0	0
**Var12**	0	0.662	−0.57	0.321	0	0

### 4.2. Hermetia illucens experiment

**Fig 3:**
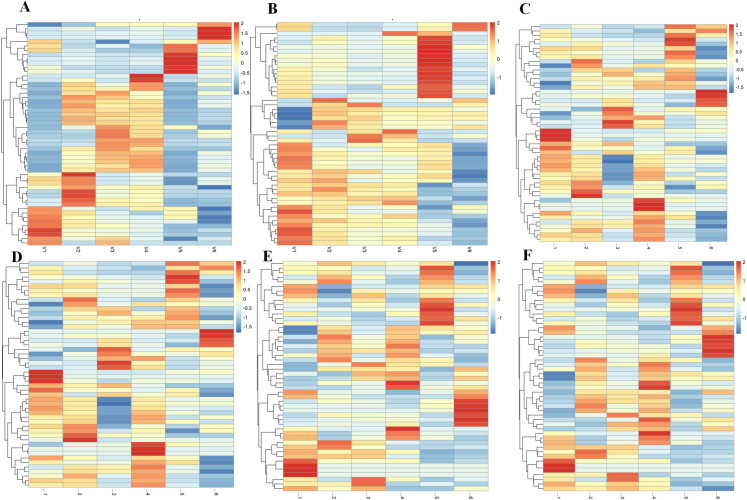
Heatmaps comparing different methods for sample classification. (A) the result of the AWGE-ESPCA model. (B) the result of the DM-ESPCA model. (C) the result of the AEs model. (D) the result of the VAEs model. (E) the result of the Lasso model. (F) the result of the Elastic Net model. The columns represent different categories, namely: Cu_0_FPKM, Cu_75_FPKM, Cu_150_FPKM. The rows are samples, and the colors in the heatmap represent the gene expression values.

The AWGE-ESPCA model achieves good results in the *Hermetia illucens* dataset. Heatmap analysis shows that the gene probes selected by AWGE-ESPCA model can better distinguish the two groups of data (Fig 3A–3C and Figs A and B in S1 Text). The heatmap generated by the AWGE-ESPCA model shows clear boundaries and significant color differences between different categories, which helps to make a clear distinction. Among other comparative models, DM-ESPCA performed best. However, the DM-ESPCA model heatmap contains a significant set of noise genes. Red indicates that the expression value of the gene probes is too large, but this group of gene probes are obviously not highly correlated with the sample distribution. In contrast, models including DM-ESPCA, SPCA, AEs, VAEs and Lasso are unable to distinguish between *Hermetia illucens* samples with low concentrations and high concentrations of Cu^2+^-sressed well. This is a good proof of our conjecture that noise in the data can significantly affect the results of feature selection.

**Fig 4:**
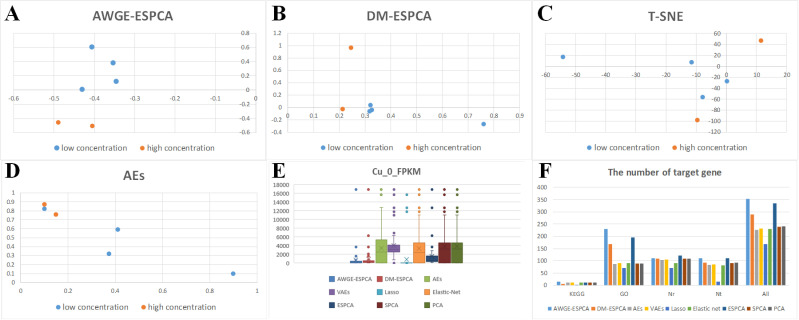
Sample distribution visualization and analysis results across different models. (A) the score plots of the AWGE-ESPCA model. (B) the score plots of the DM-ESPCA model. (C) the score plots of the T-SNE model. (D) the score plots of the AEs model. (E) boxplots comparing gene expression levels under Cu_0_FPKM condition across different models. (F) The number of target pathway genes identified by each model.

In Fig 4A–4C and Fig B in S1 Text, we observe that the AWGE-ESPCA model distinguishes the dataset with clear boundaries. Box Plots analysis (Fig 4E) further validates this advantage. In the analysis of Top-100 gene probes under Cu_0_FPKM condition, genes selected by AWGE-ESPCA show more compact distribution and fewer outliers, indicating higher inter-group differences and intra-group consistency (Fig C in S1 Text). All the unsupervised models that participated in the comparison performed poorly, particularly in the group of high-concentration samples. The probes in the two groups are far apart, making it difficult to determine whether they belong to the same group. In addition, Lasso and Elastic Net show relatively good performance in Box Plots analysis, with fewer outliers in their selected gene probes. Although Lasso and Elastic Net demonstrate relatively good control over outliers in the box plot analysis, their poor performance in gene probe selection is evident in the sample distribution plots. We attribute this to their ability to utilize grouping information through supervised learning to avoid potentially anomalous gene probes.

In pathway number analysis, AWGE-ESPCA demonstrate exceptional performance (Fig 4F and Table 2). Among the top 500 pathways with the largest loads, AWGE-ESPCA identified 353 pathways related to growth and development, with 70.4% of target genes associated with these pathways. After AWGE-ESPCA, ESPCA models follows with a performance effectiveness of 67.0%. The worst performer is the AEs model, which identify 277 pathways (with a correlation of 45.4%). The supervised models also perform less than optimally, with Lasso identifying 169 pathways (with a correlation of 33.8%) and Elastic Net identifying 229 pathways (with a correlation of 45.8%).

**Table 2:** The proportion of target pathway genes for Hermetia illucens experiment.

PCA model	The proportion of target pathway genes
**AWGE-ESPCA**	70.4%
**DM-ESPCA**	57.8%
**ESPCA**	67.0%
**SPCA**	47.8%
**PCA**	48.0%
**Lasso**	33.8%
**Elastic Net**	45.8%
**AEs**	45.4%
**VAEs**	46.2%

In summary, the results of experiment highlight the potential of AWGE-ESPCA to provide more comprehensive insights into genomic responses to Cu^2+^-stressed in *Hermetia illucens*, particularly in growth and development processes. Compared with other models, our model not only identifies more relevant pathways but also demonstrates stronger biological associations with research objectives through its adaptive noise removal and targeted gene selection capabilities.

### 4.3. Drosophilamelanogaster dataset

To verify the scalability of the AWGE-ESPCA model, we use the publicly available drosophila melanogaster dataset for verification.

**Fig 5:**
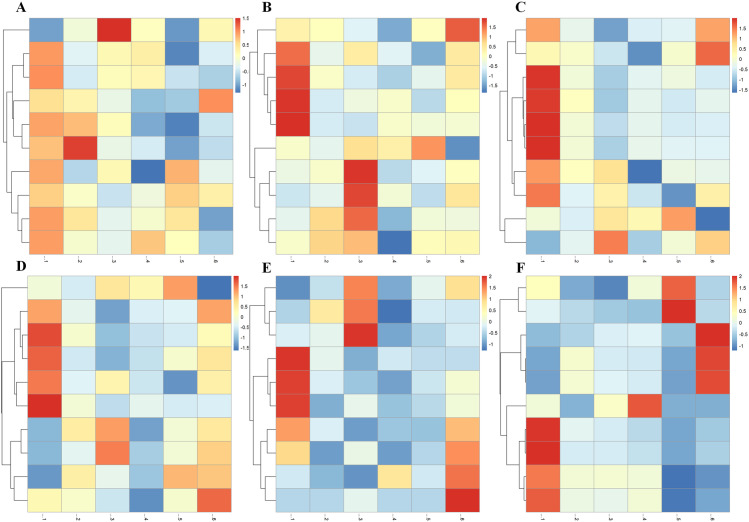
Heatmaps comparing different methods for sample classification. (A) the result of the AWGE-ESPCA model. (B) the result of the DM-ESPCA model. (C) the result of the AEs model. (D) the result of the VAEs model. (E) the result of the Lasso model. (F) the result of the Elastic Net model. The columns display two samples - P210 (P210_1, P210_2, P210_3) and T315 (T315_1, T315_2, T315_3). The rows display different genes.

**Fig 6:**
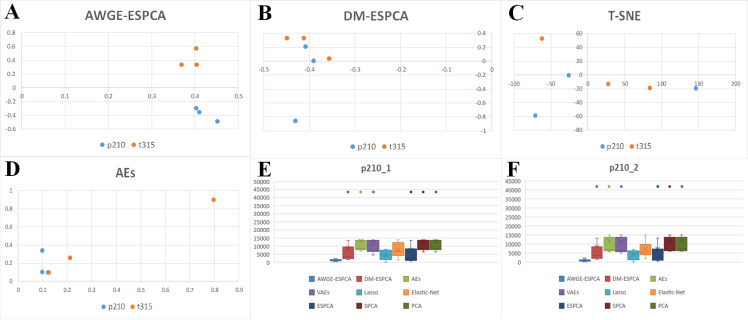
Principal component score plots and boxplots of various models. (A) The score plot of the AWGE-ESPCA model. (B) The score plot of the DM-ESPCA model. (C) The score plot of the T-SNE model. (D) The score plot of the AEs model. (E) The boxplot of the p210_1. (F) The boxplot of the p210_2.

The results are similar to the experimental results of *Hermetia illucens* dataset. AWGE-ESPCA model achieve the best experimental performance. Both heatmap analysis and sample distribution plot show that only the AWGE-ESPCA model clearly distinguished between the two groups of drosophila melanogaster samples, and the rest of the models contained an anomalous t315 group of drosophila melanogaster samples (Figs 5 and 6, and Figs D and E in S1 Text). In contrast, other unsupervised models, including DM-ESPCA and AEs, cannot get better results.

Box Plots analysis shows that only the AWGE-ESPCA model contains the smallest data, and none of the compared models can avoid choosing gene probes with larger expression values (Fig 6E and 6F, and Fig F in S1 Text). In addition, the results of the supervised models are also less than ideal, which may be due to the influence of small samples with high dimensions.

In pathway gene identification (Table 3), AWGE-ESPCA achieve an identification rate of 27.27%, which is significantly better than other models, proving that AWGE-ESPCA is still the strongest in target screening.

**Table 3:** The percentage of target pathway genes for Drosophilamelanogaster dataset.

PCA model	The proportion of target pathway genes
**AWGE-ESPCA**	27.27%
**Elastic-Net**	9.79%
**Lasso**	8.70%
**AEs**	22.8%
**VAEs**	22.8%
**DM-ESPCA**	13.64%
**ESPCA**	9.09%
**SPCA**	22.40%
**PCA**	22.40%

To summarize, all the experiments show that the AWGE-ESPCA model is superior to the existing feature extraction models, which can effectively remove the noise from insect genomic data and screen out the key gene targets and pathways.

### 4.4. Ablation experiment

To further investigate the utility of the two core modules in the AWGE-ESPCA model, Adaptive Regularization for Noise Elimination and Weighted Gene Interaction Network. We conduct ablation experiments based on the *Hermetia illucens* dataset.

We make comparisons using four metrics: sample distribution plots, number of pathways, percentage of target genes, and box plots of the distribution of expression values at gene probes. According to Table 4, the experimental results show that after the removal of the Weighted Gene Interaction Network module, the proportion of target pathway genes significantly decreased from the original 69.1% to 57.8%. In Non-Regularization models, it is 68.8%. The experimental results strongly demonstrate the role of Weighted Gene Interaction Network module in recognizing target genes.

**Table 4:** The proportion of target pathway genes for Ablation experiment.

PCA model	The proportion of target pathway genes
**AWGE-ESPCA**	69.1%
**Non-Weighted**	57.8%
**Non-Regularization**	68.8%

**Fig 7:**
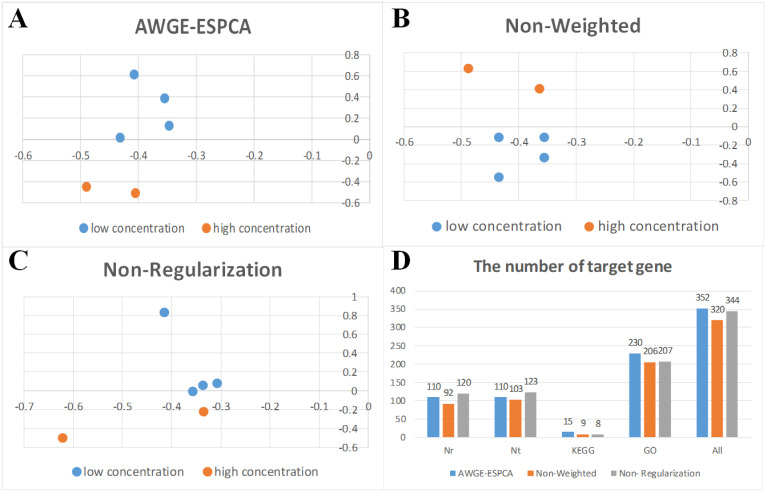
Principal Component Score Plot and the proportion of target pathway genes. (A) the result of the AWGE-ESPCA model. (B) the result of the Non-Regularization model. (C) the result of the Non-Weighted model. (D) the number of target pathway genes.

**Fig 8:**
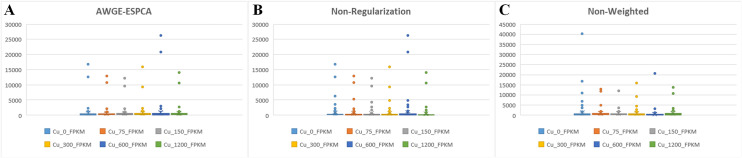
Boxplots. (A) the result of the AWGE-ESPCA model. (B) the result of the Non- Regularization model. (C) the result of the Non-Weighted model.

The sample distribution plots show that only the Non-Regularization model fails to correctly distinguish between the two groups of samples (Fig 7A–7C). This shows that Adaptive Regularization for Noise Elimination module can effectively enhance the model’s ability to discriminate between samples. Meanwhile, the number of outliers genes in the Non-Regularization model increase significantly compared to AGWE-ESPCA (Fig 8). At the same time, the performance of the Non-Weighted model is significantly better than that of Non-Regularization. These experiments strongly demonstrate the ability of Adaptive Regularization for Noise Elimination module in eliminating genomic data noise.

### 4.5. Bioenrichment analysis

**Fig 9:**
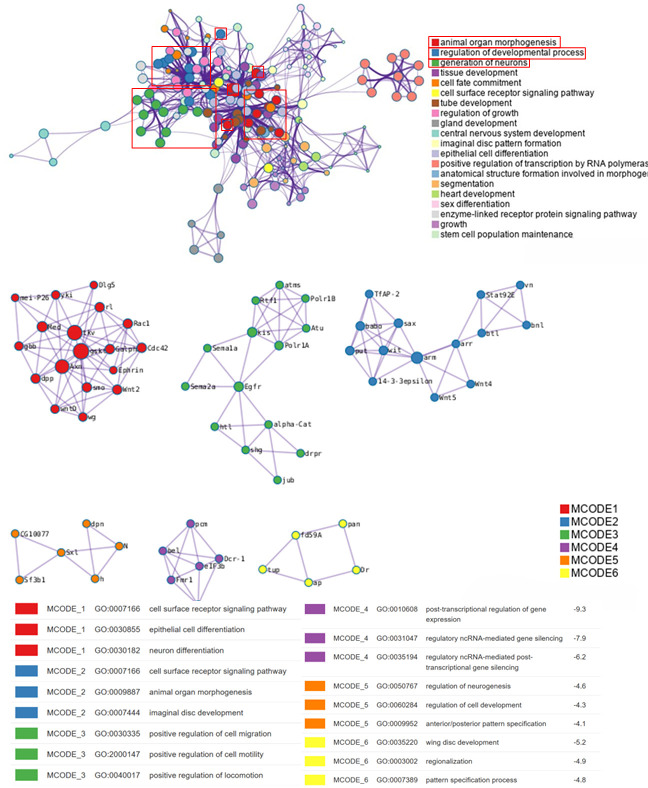
Bio-enrichment analysis picture. Bio-enrichment analysis revealing functional interactions across six MCODE modules, highlighting key pathways in cellular differentiation, neurogenesis, and morphogenesis with corresponding enrichment scores (p < 0.05).

To explore the pathway information that plays a key role in the growth of *Hermetia illucens* under Cu^2+^-stressed, we extract the PC1-Top 500 gene probes of the AWGE-ESPCA model in the *Hermetia illucens* dataset for bioenrichment analysis. We identify several key pathways including: GO:0007511, GO:0002181, GO:0006338 and GO:0032504 (Fig 9). Among them, GO:0002181 relates to protein synthesis through cytoplasmic translation, GO:0006338 is involved in chromatin structure modification, and GO:0032504 is associated with reproduction in multicellular organisms.

Among them, GO:0007511 is the pathway with the strongest correlation to the Top 500 gene probes set. Therefore, we perform a more in-depth analysis. Based on literature review and enrichment analysis, we identify FGF8, BMP2 and Notch1 gene probes as potential key sites affecting insect cardiac development.

Studies by Lewandoski, Moon, Harada and Falardeau et al. have shown that the FGF8 signaling pathway regulates the development of the midbrain and hindbrain mainly through the Otx2/Gbx2 transcription factor system [42,43], while also regulating nervous system development through the survival of GnRH neurons [45], and influencing the formation of limbs through expression in the AER region [44]. These findings suggest that Cu^2+^-stressed may affect the normal development process of multiple organ systems in the *Hermetia illucens* by disrupting the FGF8 signaling pathway.

Studies by Rivera-Feliciano, Ma and Prall et al. have shown that BMP2 regulates the fate decision of cardiac progenitor cells through the Mad/Medea transcription factor system and initiates the cardiomyocyte differentiation program through the Punt/Tkv receptor [45–47]. These findings suggest that Cu^2+^-stressed may affect the normal differentiation and development of cardiac progenitor cells by disrupting the BMP2 signaling pathway.

Studies by Boni, Urbanek and Metrich et al. have elucidated that Notch1 regulates the fate of cardiac progenitor cells through Delta/Serrate-Notch ligand-receptor interactions and regulates cardiomyocyte differentiation in a manner dependent on the Su(H) transcription factor [48–50]. These findings suggest that Cu^2+^-stressed may affect the differentiation and development of heart cells by interfering with the Notch1 signaling pathway.

In summary, the studies of MacGrogan and de la Pompa et al. show that BMP2 expression in the heart is required to maintain Notch1 expression in endothelial cells, and that these two signaling pathways regulate heart development by synergistically inducing Snail1 expression [51,52]. Meanwhile, Notch1 signaling promotes FGF8 expression in the second heart field, which in turn can remedy EMT defects [53]. These findings suggest that Cu^2+^-stressed may interfere with the heart development process of the *Hermetia illucens* by affecting the precise temporal regulatory network among these signaling pathways.

We believe that these pathways are the key pathway information for Cu^2+^-stressed to affect the growth of *Hermetia illucens*. To summarize, we demonstrate the performance of the AWGE-ESPCA model through a simulation experiment and two real datasets. The ablation experiment proves the usefulness of the Adaptive Regularization for Noise Elimination and Weighted Gene Interaction Network module proposed in this paper. In addition, in the enrichment experiments, we identify these key pathways that may affect the growth of *Hermetia illucens*. We are reasonably confident that the AWGE-ESPCA model is superior to existing Sparse PCA models, and it can provide a new artificial intelligence tool for feature extraction from insect genomic data.

## 5. Discussion

In the process of studying the effects of Cu^2+^-stressed on *Hermetia illucens*, we delve into several significant challenges faced in the genomic analysis of *Hermetia illucens*. The main issues include the scarcity of high-quality genomic data, noise in genomic data, and the inability of existing models to select pathway-enriched regions well. It is important to note that, unlike human gene data sets, the sample size of insect genomic data set is particularly small. Therefore, it is difficult to use supervised models (e.g., Lasso or Elastic Net) to build a classification model with sufficient robustness and generalization performance. The same is true for the choice of regularization models, and $L_{1}$ and $L_{2}$ regularizers are widely used in the genomics field for dimensionality reduction and feature selection with good results. However, this type of regularizer requires supervised operations. Therefore, it is difficult to obtain good enough performance in such small sample datasets. This is also verified in our experiments. We make progress in overcoming these challenges by developing and applying the AWGE-ESPCA model.

Firstly, we design experiments and construct a genomic dataset containing six samples of Cu^2+^ stressed *Hermetia illucens* growth. This dataset allows us to study how *Hermetia illucens* larvae are affected by Cu^2+^-stressed during growth resulting in growth differences. This is essential to enhance the safety of *Hermetia illucens* as feed. Secondly, for the noisy characteristics of the *Hermetia illucens* genomic dataset, we propose a novel Adaptive Regularization for Noise Elimination and integrate it into the existing Sparse PCA framework. The $L_{AR}$ regularizer can adaptively remove gene probes with excessive expression values in the *Hermetia illucens* genomic data. Our experimental analysis demonstrates that gene probes exhibiting unusually high expression values frequently lack discriminatory power between different sample groups. The $L_{AR}$ regularizer proves to be more efficient and flexible than traditional manual data correction approaches in addressing this challenge. To validate whether the eliminated gene probes represent important biological signals, we conduct enrichment analysis on the potential noise gene probe set (1037 gene probes). The results show that 58.8% of gene probes are not associated with any known pathways, 35.2% are associated with only one pathway, and merely 6% are associated with multiple pathways. Furthermore, P-value analysis reveal that only 10.41% of these genes show statistical significance (P < 0.05), with an average P-value of 0.42, suggesting these eliminated probes are more likely to represent random fluctuations rather than biologically significant signals (Fig G in S1 Text). Thirdly, we propose the Weighted Gene Interaction Network. This enables the model to focus more on regions within the gene network that are rich in pathways, which are crucial in biological processes. In addition, we would like to discuss the running time, memory consumption and scalability of the model in particular. All our experiments are performed on a computer with an AMD 7950X CPU and 32GB RAM. We compare the running time and memory consumption of the AWGE-ESPCA model with the comparison model on the *Hermetia illucens* dataset (Table B in S1 Text). The experiment shows that researchers do not need expensive computing equipment to use the AWGE-ESPCA model (42 min/520 MB). We also test the AWGE-ESPCA model on a large genomic dataset (100 samples × 25,000 genes), which requires approximately 4.5 hours of processing time and 3.8 GB memory usage. This supplementary experiment shows that the AWGE-ESPCA model has good scalability. Theoretically, the AWGE-ESPCA model can be applied to the analysis of even larger and more complex human and other biological datasets.

Although the experimental results prove the superiority of AWGE-ESPCA model, this study still has the following two limitations. First, the sample size of the genomic dataset constructed in this study for Cu^2+^-stressed *Hermetia illucens* growth is still small, which may affect the accuracy of the results. Second, the model only considers short-connected biological pathway information, i.e., pairwise pathway relationships, when performing feature selection. However, it is clear that researchers will like the model to select long pathway information that is as complete as possible, which will facilitate further wet experimental design. In the future, we plan to further design experiments to supplement the genomic dataset of Cu^2+^-stressed *Hermetia illucens* growth and continue to improve the AWGE-ESPCA model to enhance its feature selection capability. In summary, we propose an artificial intelligence feature extraction model specifically designed for *Hermetia illucens* genome analysis. Experiments confirm the superior performance of the AWGE-ESPCA model. In addition, we confirm that the AWGE-ESPCA model can be effectively extended to other insect genome analysis tasks. We believe that the AWGE-ESPCA model can help researchers identify potential biomarkers in insect genomes more efficiently.

## Supporting information

S1 Text Supplementary information. **Fig A. Heatmaps of the ESPCA, SPCA, PCA model.** (A) the result of the ESPCA model. (B) the result of the SPCA model. (C) the result of the PCA model. **Fig B. Principal component score plots of UMAP, VAEs, ESPCA, SPCA, PCA model.** (A) the score plots of the UMAP model. (B) the score plots of the VAEs model. (C) the score plots of the ESPCA model. (D) the score plots of the SPCA model. (E) the score plots of the PCA model. **Fig C. Boxplots of Cu_75_FPKM, Cu_150_FPKM, Cu_300_FPKM, Cu_600_FPKM, Cu_1200_FPKM.** (A) the boxplots of the Cu_75_FPKM. (B) the boxplots of the Cu_150_FPKM. (C) the boxplots of the Cu_300_FPKM. (D) the boxplots of the Cu_600_FPKM. (E) the boxplots of the Cu_1200_FPKM. **Fig D. Heatmaps of the ESPCA, SPCA, PCA model.** (A) the result of the ESPCA model. (B) the result of the SPCA model. (C) the result of the PCA model. **Fig E. Principal component score plots of UMAP, VAEs, ESPCA, SPCA, PCA models.** (A) the result of the UMAP model. (B) the result of the VAEs model. (C) the result of the ESPCA model. (D) the result of the SPCA model. (E) the result of the PCA model. **Fig F. Boxplots comparing gene expression levels between P210 and T315I samples.** (A) the boxplots for P210 replicate 3. (B) the boxplots for T315I replicate 1. (C) the boxplots for T315I replicate 2. (D) the boxplots for T315I replicate 3. **Fig G. Noise probes correlation analysis and pathway number analysis plots.** (A) Distribution of P_value for probes included in the noisy probes. (B) Distribution of the number of individual gene probes associated with known pathways in the noisy gene probes. **Table A. The top two identified PC1 and PC2 loadings by SPCA and PCA. Table B. Time and memory usage comparison across methods.**
(DOCX)
