# Supplementary material for: AWGE-ESPCA: An edge sparse PCA model based on adaptive noise elimination regularization and weighted gene network for Hermetia illucens genomic data analysis
Source: PLoS Comput Biol. 2025 Feb 13;21(2):e1012773. doi: 10.1371/journal.pcbi.1012773 (PMC11825005; doi:10.1371/journal.pcbi.1012773)
Supplement: S1 Text — Fig A. Heatmaps of the ESPCA, SPCA, PCA model. (A) the result of the ESPCA model. (B) the result of the SPCA model. (C) the result of the PCA model. Fig B. Principal component score plots of UMAP, VAEs, ESPCA, SPCA, PCA model. (A) the score plots of the UMAP model. (B) the score plots of the VAEs model. (C) the score plots of the ESPCA model. (D) the score plots of the SPCA model. (E) the score plots of the PCA model. Fig C. Boxplots of Cu_75_FPKM, Cu_150_FPKM, Cu_300_FPKM, Cu_600_FPKM, Cu_1200_FPKM. (A) the boxplots of the Cu_75_FPKM. (B) the boxplots of the Cu_150_FPKM. (C) the boxplots of the Cu_300_FPKM. (D) the boxplots of the Cu_600_FPKM. (E) the boxplots of the Cu_1200_FPKM. Fig D. Heatmaps of the ESPCA, SPCA, PCA model. (A) the result of the ESPCA model. (B) the result of the SPCA model. (C) the result of the PCA model. Fig E. Principal component score plots of UMAP, VAEs, ESPCA, SPCA, PCA models. (A) the result of the UMAP model. (B) the result of the VAEs model. (C) the result of the ESPCA model. (D) the result of the SPCA model. (E) the result of the PCA model. Fig F. Boxplots comparing gene expression levels between P210 and T315I samples. (A) the boxplots for P210 replicate 3. (B) the boxplots for T315I replicate 1. (C) the boxplots for T315I replicate 2. (D) the boxplots for T315I replicate 3. Fig G. Noise probes correlation analysis and pathway number analysis plots. (A) Distribution of P_value for probes included in the noisy probes. (B) Distribution of the number of individual gene probes associated with known pathways in the noisy gene probes. Table A. The top two identified PC1 and PC2 loadings by SPCA and PCA. Table B. Time and memory usage comparison across methods. (DOCX) [file pcbi.1012773.s001.docx]

Supplementary Information

AWGE-ESPCA: An Edge Sparse PCA Model Based on Adaptive Noise Elimination Regularization and Weighted Gene Network for Hermetia illucens genomic data analysis

${Rui Miao}^{1+}$, Hao-Yang $\mathrm{Yu}^{1+}$, Bing-Jie $\mathrm{Zhong}^{1}$, Hong-Xia $\mathrm{Sun}^{1}$,Qiang $\mathrm{Xia}^{1}$^[[1]](#footnote-1)^

1. Basic Teaching Department, Zhuhai Campus of Zunyi Medical University, Zhu Hai, China.

*Corresponding author

E-mail Address: xiaqiang1973@126.com

**1. Evaluation indicators**

**1.1 Heatmap (PC1 Top-50)**

A heatmap is a graphical representation of data where individual values are represented as colors. For the indicator "Heatmap (PC1 Top-50)," the heatmap visualizes the top 50 principal components (PC1) based on their significance or variance in the dataset. This allows for the identification of patterns, correlations, and clusters within the data, providing insights into the distribution and relationships of the top 50 features under PC1. The use of a heatmap for this purpose aids in visualizing complex data matrices in a more comprehensible manner.

**1.2 Sample Distribution Plot (based on PC1 and PC2)**

A sample distribution plot based on the first two principal components (PC1 and PC2) visually represents the spread and clustering of samples in the reduced dimensional space. It helps in identifying clusters, outliers, and potential separability of different groups or conditions within the dataset. This plot primarily aims to clearly present the results of dimensionality reduction, allowing for a clear understanding of the PCA model's specific differentiation of different classes.

**1.3 Number of Pathways (PC1 Top-500)**

The "Number of Pathways" indicator reflects the count of biological pathways that are significantly associated with the top 500 genes or features identified under the first principal component (PC1). This metric is important for understanding the biological relevance and functional implications of the most significant features. By analyzing the number of pathways, researchers can gain insights into the underlying biological processes, mechanisms, and potential targets for further study or therapeutic intervention.

**1.4 Target Gene Percentage (genes/total number of genes with experimental target function)**

The "Target Gene Percentage" measures the proportion of genes with an experimental target function relative to the total number of genes. This indicator provides insights into the specificity and relevance of the dataset or model in capturing genes that have known functional roles or experimental validation. It helps in evaluating the enrichment and representation of target genes within the dataset, thereby assessing the biological validity and potential impact of the findings.

**1.5 Box Plots of Gene Probe Expression (PC1 Top-100)**

Box plots are a method for graphically depicting groups of numerical data through their quartiles. For the indicator "Box Plots of Gene Probe Expression (PC1 Top-100)," box plots are used to visualize the distribution and variability of gene expression levels for the top 100 principal components (PC1). This method helps in identifying outliers, comparing distributions across different conditions or groups, and understanding the central tendency and dispersion of gene expression data. Box plots are valuable for summarizing complex datasets and facilitating comparisons in a visually intuitive manner.

**2. Supplementary experiment**

**2.1 The Experiment of Noise Set Construction and Validation**

To verify whether adaptive regularizers incorrectly remove a large number of important gene probes or miss important pathway information, we designed a systematic analysis and validation scheme. First, based on the hermetia illucens dataset, we construct an experiment to determine the information of gene probes removed by the AWGE-ESPCA model in PC1 and construct a set of potential noise gene probes (1037 gene probes). Next, we perform enrichment analysis for this noisy gene probes set with the aim of exploring whether there are key targets and corresponding pathway information related to the growth and development of the hermetia illucens in the gene probes that are considered as noise by the model. Finally, we also perform P_value analysis for the noisy gene set with the aim of confirming the percentage of gene probes that are highly correlated with insect grouping information.

**2.2 The Experiment of Computational Performance Evaluation**

To comprehensively evaluate the computational efficiency and algorithmic scalability of the AWGE-ESPCA model, we designed and implemented a systematic performance analysis framework. The experimental platform was configured with an RTX 4060Ti GPU, AMD 7950X CPU, and 32GB RAM architecture, utilizing the Hermetia illucens genomic dataset as our evaluation benchmark. The performance analysis framework compared AWGE-ESPCA with four established methodologies: DM-ESPCA, ESPCA, Elastic Net, and Lasso. We evaluated computational runtime and memory utilization metrics for each approach, enabling systematic assessment of algorithmic efficiency and resource requirements across genomic data analysis tasks.

**3. Supplementary figure**

**3.1 heatmap based on hermetia illucens datasets**

**Fig A**. **Heatmaps of the ESPCA, SPCA, PCA model.** (A) the result of the ESPCA model. (B) the result of the SPCA model. (C) the result of the PCA model.

**3.2 Principal component score plots on hermetia illucens datasets**

**Fig B. Principal component score plots of UMAP, VAEs, ESPCA, SPCA, PCA model.** (A) the score plots of the UMAP model. (B) the score plots of the VAEs model. (C) the score plots of the ESPCA model. (D) the score plots of the SPCA model. (E) the score plots of the PCA model.

**3.3 boxplots based on hermetia illucens datasets**

**Fig C.** **Boxplots of Cu_75_FPKM, Cu_150_FPKM, Cu_300_FPKM, Cu_600_FPKM, Cu_1200_FPKM.** (A) the boxplots of the Cu_75_FPKM. (B) the boxplots of the Cu_150_FPKM. (C) the boxplots of the Cu_300_FPKM. (D) the boxplots of the Cu_600_FPKM. (E) the boxplots of the Cu_1200_FPKM.

**3.4 heatmap based on Drosophilamelanogaster datasets**

**Fig D. Heatmaps of the ESPCA, SPCA, PCA model.** (A) the result of the ESPCA model. (B) the result of the SPCA model. (C) the result of the PCA model.

**3.5 Principal component score plots based on Drosophilamelanogaster datasets**

**Fig E. Principal component score plots of UMAP, VAEs, ESPCA, SPCA, PCA models.** (A) the result of the UMAP model. (B) the result of the VAEs model. (C) the result of the ESPCA model. (D) the result of the SPCA model. (E) the result of the PCA model.

**3.6 Boxplots comparing gene expression levels between P210 and T315I samples**

**Fig F. Boxplots comparing gene expression levels between P210 and T315I samples.** (A) the boxplots for P210 replicate 3. (B) the boxplots for T315I replicate 1. (C) the boxplots for T315I replicate 2. (D) the boxplots for T315I replicate 3.

**3.7 Noise probes correlation analysis and pathway number analysis plots**

**Fig G. Noise probes correlation analysis and pathway number analysis plots.** (A) Distribution of P_value for probes included in the noisy probes. (B) Distribution of the number of individual gene probes associated with known pathways in the noisy gene probes.

**4. Supplementary table**

**4.1 The top two identified PC1 and PC2 loadings by PCA and SPCA**

**Table A. The top two identified PC1 and PC2 loadings by SPCA and PCA**

| Method | SPCA | | PCA | |
| --- | --- | --- | --- | --- |
| PC | PC1 | PC2 | PC1 | PC2 |
| Var1 | -0.001 | -0.006 | 0.001 | 0.0032 |
| Var2 | 0 | 0 | 0 | 0.041 |
| Var3 | 0 | 0.028 | 0.001 | 0.043 |
| Var4 | 0 | 0 | -0.001 | 0.045 |
| Var5 | 0 | 0 | 0 | 0.012 |
| Var6 | -0.002 | -0.052 | 0.001 | -0.033 |
| Var7 | 0 | 0 | 0.001 | -0.029 |
| Var8 | 0 | 0 | 0.001 | -0.009 |
| Var9 | -0.568 | 0 | 0.494 | 0.322 |
| Var10 | -0.587 | -0.525 | 0.511 | -0.838 |
| Var11 | -0.577 | 0.531 | 0.502 | 0.41 |
| Var12 | 0 | 0.662 | 0.493 | 0.128 |

**4.2 Time and Memory Usage Comparison Across Methods**

**Table B. Time and Memory Usage Comparison Across Methods**

| **Metric** | **AWGE-ESPCA** | **DM-ESPCA** | **ESPCA** | **Elastic Net** | **Lasso** |
| --- | --- | --- | --- | --- | --- |
| **Time** | 42min | 28min | 26min | 2.8s | 2.1s |
| **Memory** | 520MB | 490MB | 380MB | 280MB | 220MB |

1. [↑](#footnote-ref-1)
